# Increased circulating bioactive C-type natriuretic peptide is associated with reduced heart rate variability in patients with chronic kidney disease

**DOI:** 10.1186/s12882-018-0843-3

**Published:** 2018-03-05

**Authors:** Lulu Wang, Wenjin Liu, Yanting Yu, Lei Jiang, Junwei Yang

**Affiliations:** 1 Center for Kidney Disease, Second Affiliated Hospital of Nanjing Medical University, 262# North Zhongshan Road, Nanjing, 210003 China; 2 Departments of nephrology, BenQ Medical Center, The Affiliated BenQ Hospital of Nanjing Medical University, Nanjing, China

**Keywords:** CNP, Natriuretic peptide, CKD, Cardiovascular disease, Heart rate variability

## Abstract

**Background:**

C-type natriuretic peptide (CNP) is a member of the natriuretic peptide family and have been implicated to be involved in maintaining vascular homeostasis and acting as a cardiac chronotropic agent in experimental studies. However, clinical evidence of its participation in cardiovascular regulation is lacking, especially in patients with chronic kidney disease (CKD). We aimed to explore the association of circulating CNP with cardiovascular alterations in CKD.

**Methods:**

Seventy-six subjects with CKD were recruited. Plasma CNP-22, the bioactive form of CNP in the circulation, was measured by an enzyme immunoassay. The patients also underwent several cardiovascular evaluations including measurement of blood pressure, endothelial function, heart rate variability (HRV) and pulse wave velocity.

**Results:**

Mean (±standard deviation) age of the patients were 59.9 (±14.9) years and 56.6% were male. Average plasma CNP level was 790.8 ± 309.1 pg/ml. Plasma CNP level was not increased as estimated glomerular filtration rate declined. There was no significant difference of CNP between patients with or without endothelial dysfunction (with vs. without endothelial dysfunction: 844.6 ± 365.5 pg/ml vs. 738.3 ± 231.8 pg/ml, *p* = 0.14). Plasma CNP showed no association with blood pressure or pulse wave velocity, but was negatively associated with time-domain HRV parameters (SDNN, RMSSD, Triangular Index). The association of CNP with HRV persisted after adjustment for potential covariates.

**Conclusions:**

Our data highlights a possible link between circulating CNP and autonomic dysfunction in CKD patients. Further studies are warranted to explore the mechanisms underlying this association, as well as evaluate the ability of circulating CNP in predicting adverse cardiovascular event in CKD patients.

**Electronic supplementary material:**

The online version of this article (10.1186/s12882-018-0843-3) contains supplementary material, which is available to authorized users.

## Background

Chronic kidney disease (CKD) is a well-known potent risk factor for developing cardiovascular disease [1, 2]. Individuals with CKD are more likely to die from cardiovascular causes than to progress to end-stage renal disease [3]. The mechanisms underpinning this increased cardiovascular risk in CKD are complex and remain to be fully elucidated. A variety of non-traditional risk factors, as well as traditional ones, have been recognized to be involved in the pathogenesis of cardiovascular injury in CKD [4]. An extensive understanding of the non-traditional factors participating in the modulation of cardiovascular system may therefore provide novel strategy for cardiovascular protection in CKD patients.

C-type natriuretic peptide (CNP) is a cardiovascular bioactive hormone of the natriuretic peptide family and shares structure homology with the other two cardiac peptide, atrial natriuretic peptide (ANP) and B-type natriuretic peptide (BNP) [5]. It is encoded by the *Nppc* gene on chromosome 2 and is most widely expressed in the brain and endothelial cells [6]. It is synthesized as a 126 aa peptide (Prepro-CNP) and is converted to the NT-proCNP by removal of the signal peptide. The NT-proCNP is cleaved by furin to yield CNP-53, which is then processed further to yield the bioactive form, CNP-22 [5]. Previous work investigating the biological function of CNP has revealed an important role for it in maintaining vascular homeostasis and blood pressure regulation [7, 8]. It can also act as a cardiac inotropic and chronotropic agent [9, 10]. To be noted, the biological activity of CNP are thought to be exerted in a paracrine or autocrine fashion due to the low circulating level. However, recent clinical studies have identified alteration of plasma CNP level in patients with heart failure [11, 12] and have demonstrated a link between elevated plasma CNP and increased risk of myocardial infarction in the general population [13]. These data indicate that circulating CNP is also involved in cardiovascular modulation and diseases.

Although ANP and BNP have been extensively studied in patients with kidney disease, there is a paucity of data regarding CNP and its relationship to cardiovascular alteration in CKD. Questions remain to be answered include: (1) is plasma CNP accumulated as renal function declines? (2) is there any association of circulating bioactive CNP-22 with cardiovascular alteration in CKD patients? To answer the questions, we conducted the current cross-sectional study. We measured plasma CNP-22 in a group of non-dialysis-dependent CKD patients and explore its association with (1) estimated glomerular filtration rate (eGFR) and (2) several cardiovascular measures which were chosen based on prior experimental evidence, including blood pressure, endothelial function, arterial elasticity and heart rate variability (HRV).

## Methods

### Study subjects

All participants were recruited from the Center for Kidney disease of the Second Affiliated Hospital of Nanjing Medical University. Patients aged over 18 years with a clinical diagnosis of CKD referring to our center were invited to participate in the study. The diagnoses of CKD were made by clinical physicians conforming to the definition of K/DOQI guideline and had been confirmed by study staffs at enrollment. Exclusion criteria were: (1) previous history of renal transplantation; (2) malignancy; (3) acute infection or any other unstable condition. All subjects provided written informed consents. The study protocol was approved by the Institutional Ethical Committee of the Second Affiliated Hospital of Nanjing Medical University.

### Cardiovascular evaluations

All cardiovascular evaluations were performed in the morning with the patients on an empty stomach. Antihypertensive medications and nitrates were not allowed within 2 h, and long-acting nitrates were not permitted for 12 h before measurement. Test room temperature was set at 22-25 °C.

Patients rested for 10 min in a seated position and then underwent three consecutive blood pressure measurements using an automated oscillometric device (Omron HEM-7130; Omron Healthcare Co. Ltd., Kyoto, Japan). Each measurement was separated by 1 min interval. The three measurements were averaged to calculate blood pressure for final analysis.

Carotid–femoral pulse wave velocity (cfPWV) and carotid–radial pulse wave velocity (crPWV) were determined after blood pressure measurement. An experienced technician performed the measurement for all participants using the Complior Analyzer device (Artech Medical; Paris, France). Three probes were placed in a place of palpable pulse of the carotid, femoral and radial artery, respectively. The transit time was averaged over ten consecutive recordings using the intersecting tangent algorithm as recommended [14]. Carotid–femoral and carotid–radial distances were measured and multiplied by 0.8 for calculation of pulse wave velocity.

Endothelial function and HRV were measured via peripheral arterial tonometry using the EndoPAT 2000 (Itamar Medical Inc., Israel). Patients underwent the test in seated position with two finger probes placed on the index finger of each hand. After 6 min of baseline measurement, pulsatile arterial flow was occluded through inflation of a blood pressure cuff on the test arm for 5 min. The cuff was inflated starting at a pressure of 250 mmHg and increased until a complete occlusion was achieved as judged by the PAT signal or to a maximum of 300 mmHg. After occlusion, PAT signal was recorded for another 5 min. Reactive hyperemia index (RHI) were calculated automatically by the system [15]. Endothelial dysfunction is defined as RHI < 1.67. HRV indices including SDNN (standard deviation of the normal-to-normal interval), RMSSD (the square root of the mean squared differences of successive normal-to-normal intervals), Triangular Index and LF/HF (the ratio of low frequency to high frequency components) is calculated from the baseline period.

### Plasma CNP

Fasting blood were drawn into a EDTA tube to separate plasma. All sample were aliquoted and stored at − 80 °C until measurement. Plasma CNP was detected by an enzyme immunoassay kit (EKE-012-03, Phoenix Pharmaceuticals, Inc. California, USA). This assay detected the bioactive form of CNP (i.e CNP-22) with a range from 0 to 100 ng/ml. The coefficient of variation at the level of 100 pg/ml was 16.6%.

### Clinical information

Demographic and medical information (medical history and medications) were acquired by a combination of patient interview and review of medical records. Previous history of cardiovascular disease refers to any history of acute myocardial infarction, ischemic or hemorrhagic stroke, coronary heart disease other than myocardial infarction, chronic heart failure, atrial fibrillation and other types confirmed by the research staff. All routine laboratory tests were performed in the local Laboratory Department using fasting blood or first voided morning urine. Estimated glomerular filtration rate (eGFR) was calculated using the CKD-EPI formula. CKD stage classification were determined as recommended by the K/DOQI guideline [16].

### Statistical analyses

Data were presented as the mean ± standard deviation or the median (interquartile range) for numerical variables, and as counts (%) for categorical variables. Comparisons of CNP levels between groups were performed using Student’s t test or the chi-square test as appropriate. To evaluate the relationship of plasma CNP with cardiovascular measures, Pearson’s correlation analysis was used. Due to the skewed distribution of HRV parameters, there were logarithm transformed in these analyses. To confirm the association of CNP with HRV, multiple linear regression analyses were conducted with adjustment for several potential covariates, including age, sex, eGFR, diabetes mellitus, body mass index, current smoker, previous history of cardiovascular diseases, hemoglobin and systolic blood pressure. For the selection of covariates, we first checked the associations of all available factors with HRV parameters and found none of them has a consistent association with HRVs (“consistent” defined as significantly associated with at least two HRV parameters) except for sex. Considering the small sample size, our choices of covariates were then mainly determined by whether there is known or expected association with the outcome variable (HRV) which is justified on the basis of previous evidence [17]. All analyses were performed using SPSS 19.0 (IBM SPSS, Chicago, IL). Figures were generated by GraphPad Prism 6.0 (GraphPad Software Inc., San Diego, CA). A two-tailed *p* value < 0.05 was considered statistically significant.

## Results

**Fig. 1:**
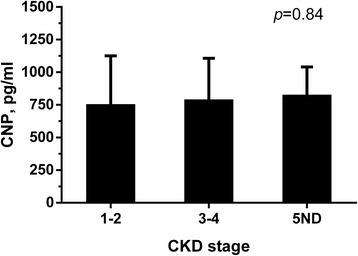
Plasma CNP levels in different CKD stages. 5ND indicates CKD stage-5 (non-dialysis)

A total of 76 patients participated in the current study. General characteristics of the study patients are presented in Table 1. Mean (±standard deviation) age of the patients were 59.9 (±14.9) years and 56.6% were male. Diabetic kidney disease and glomerulonephritis were the leading causes of CKD in these patients. Mean eGFR was 41.4 (±25.4) ml/min/1.73m^2^ and 42.1% patients had a previous history of cardiovascular disease. Average plasma CNP level was 790.8 ± 309.1 pg/ml. To evaluate whether renal dysfunction is associated with altered circulating CNP level, we compared plasma CNP concentration in different CKD stages as shown in Fig. 1. Mean plasma CNP was 755.0 (±370.7) pg/ml, 791.9 (±314.7) pg/ml and 828.2 (±211.9) pg/ml in patients with stage 1-2 CKD, stage 3-4 CKD and stage 5ND CKD, respectively. There was no significant difference between each group (*p* = 0.84).

**Table 1:** General information of the study patients

	Mean ± SD / Median (IQR) or N (%)
Age, years	59.9 ± 14.9
Male	43 (56.6%)
BMI, kg/m^2^	24.7 ± 3.7
Current smoker	20 (26.3%)
Etiology	
Diabetic kidney disease	22 (28.9%)
Hypertensive nephropathy	6 (7.9%)
Glomerulonephritis	18 (23.7%)
Others	7 (9.2%)
Undetermined	23 (30.3%)
Previous CVD	32 (42.1%)
Myocardial infarction	3 (3.9%)
Stroke	27 (35.5)
Coronary heart disease	6 (7.9%)
Atrial fibrillation	2 (2.6%)
eGFR, mL/min/1.73m^2^	41.4 ± 25.4
Hemoglobin, g/L	112.5 ± 22.5
Albumin, g/L	36.0 ± 6.9
Total cholesterol, mmol/L	5.18 ± 2.03
Triglycerides, mmol/L	1.89 ± 1.52
HDL-C, mmol/L	1.23 ± 0.46
LDL-C, mmol/L	3.06 ± 1.23
Calcium, mmol/L	2.17 ± 0.20
Phosphorus, mmol/L	1.17 ± 0.23
CRP, mg/L	1.1 (0.5 - 4.0)
Urine PCR, mg/mmol	246.8 (55.6 – 493.3)
CNP, pg/ml	790.8 ± 309.1

*Abbreviations*: *BMI* Body mass index, *CNP* C-type natriuretic peptide, *CRP* C-reactive protein, *CVD* Cardiovascular disease, *eGFR* Estimated glomerular filtration rate, *HDL-C* High-density lipoprotein cholesterol, *LDL-C* Low-density lipoprotein cholesterol, *PCR* Protein to creatinine ratio

**Fig. 2:**
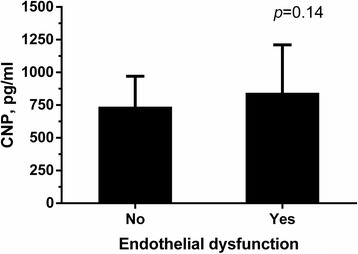
Plasma CNP levels in patients with or without endothelial dysfunction

Endothelial cells are the primary source of circulating CNP in humans. We therefore explored whether patients with endothelial dysfunction exhibit reduced plasma CNP level (Fig. 2). The results indicate that plasma CNP level was comparable between patients with or without endothelial dysfunction (with vs. without endothelial dysfunction: 844.6 ± 365.5 pg/ml vs. 738.3 ± 231.8 pg/ml, *p* = 0.14).

Cardiovascular evaluation results are presented in Table 2 and their relationships to plasma CNP was evaluated by Pearson’s correlation analysis. CNP showed no significant correlation with systolic or diastolic blood pressure, cfPWV or crPWV and RHI, whereas it was correlated positively with heart rate (*r* = 0.23, *p* = 0.04) and negatively with SDNN (*r* = − 0.33, *p* = 0.004), RMSSD (*r* = − 0.27, *p* = 0.02) and triangular index (*r* = − 0.33, *p* = 0.005) (all HRV parameters were logarithm transformed in Pearson’s correlation analyses).

**Table 2:** Cardiovascular evaluation results and their relationship with plasma CNP

	Mean ± SD or Median (IQR)	*r*	*p*
Systolic BP	135.8 ± 20.7	−0.04	0.74
Diastolic BP	83.7 ± 11.5	0.06	0.64
Heart rate	74.8 ± 12.2	0.23	**0.04**
cfPWV	11.4 ± 4.3	−0.04	0.71
crPWV	10.0 ± 3.4	−0.11	0.34
RHI	1.90 ± 0.49	−0.05	0.69
SDNN^a^	30.8 (19.8 – 57.6)	−0.33	**0.004**
RMSSD^a^	30.5 (17.2 – 56.3)	−0.27	**0.02**
Triangular Index^a^	8.5 (5.7 – 12.0)	−0.33	**0.005**
LF/HF^a^	0.63 (0.32 – 0.89)	0.04	0.75

^a^These parameters were logarithm transformed in Pearson’s correlation analysis due to their skewed distribution

Bold entries indicate statistical significance

*Abbreviations*: *BP* Blood pressure, *cfPWV* Carotid–femoral pulse wave velocity, *crPWV* Carotid–radial pulse wave velocity, *IQR* Interquartile range, *RHI* Reactive hyperemia index

The correlation analysis gave a clue that circulating CNP is associated with altered HRV, rather than vascular homeostasis indices in CKD patients. We hence sought to confirm the associations between CNP and HRV parameters using multiple linear regression analysis with adjustment for several covariates, including age, sex, eGFR, diabetes mellitus, body mass index, current smoker, previous history of cardiovascular diseases, hemoglobin and systolic blood pressure. As shown in Table 3, CNP remained significantly associated with logarithm transformed SDNN, RMSSD and triangular index after adjustment (SDNN: ß = − 0.12, *p* = 0.003; RMSSD: ß = − 0.11, *p* = 0.02; Triangular index: ß = − 0.08, *p* = 0.004; all ß were calculated per SD increment of CNP). In a sensitivity analysis excluding patients with coronary artery disease (*n* = 9), the associations remained essentially unchanged (Additional file 1: Table S1).

**Table 3:** Associations between plasma CNP and HRV parameters

	Plasma CNP		
	ß	95% confidence interval	*p*
SDNN^a^	− 0.12	−0.19 – −0.04	**0.003**
RMSSD^a^	− 0.11	−0.20 – −0.02	**0.02**
Triangular Index^a^	− 0.08	−0.14 – −0.03	**0.004**
LF/HF^a^	0.02	−0.08 – 0.13	0.65

^a^These parameters were logarithm transformed in the analysis due to their skewed distribution

ß indicates per SD increment of CNP and was adjusted for age, sex, eGFR, diabetes mellitus, body mass index, current smoker, previous history of cardiovascular diseases, hemoglobin and systolic blood pressure

Bold entries indicate statistical significance

## Discussions

In the current study, we explored the associations of plasma bioactive CNP with renal function and the cardiovascular system in a group of CKD patients. Our major findings are as following: (1) Increased plasma CNP was associated with reduced HRV; (2) Decreased renal function was not associated with elevated plasma CNP level; (3) Circulating CNP was not reduced in patients with endothelial dysfunction compared to those with preserved endothelial function; (4) There was no association between plasma CNP and blood pressure or arterial elasticity (neither central or peripheral).

Besides several negative results, we found a significant association of increased plasma CNP with elevated heart rate and reduced HRV. The positive association of CNP with heart rate is in accordance with the chronotropic effect of CNP found in both clinical and experimental settings. Cheung et al. also noted a significant correlation between CNP and heart rate in hypertensive subjects [18]. Moreover, they measured plasma noradrenaline and found a positive association of plasma CNP with noradrenaline. In the study by Igaki et al., intravenous administration of CNP induced an increase of heart rate in healthy subjects [19]. The work by Beaulieu et al. shed light on the underlying mechanism as they found that CNP can increase the maximal depolarization rate during diastole and decrease the action potential duration of repolarization in dogs [10].

The significant and negative association between CNP and HRV in the patients as revealed by our data is very interesting. Since reduced HRV has been proved to be an independent risk factor for cardiovascular events [20], our data may therefore provide insights into the underpinning mechanism of the link between elevated circulating CNP and increased cardiovascular risk in the general population [13]. To the best of our knowledge, our data for the first time show a relationship between CNP and altered HRV in humans and can be interpreted in combination with data from previous experimental settings. In a recent study using a transgenic rat model with neuron-specific overexpression of a dominant negative NPR-B receptor, Buttgereit et al. showed that CNP signaling via the NPR-B receptor is sympathoinhibitory [21]. Noteworthy,overall HRV as assessed by SDNN was reduced in the transgenic rats while LF/HF was increased. In addition, Moyes et al. found increased LF/HF in female endothelial-specific CNP knock-out mouse with no significant change in SDNN [7]. We observed a negative association of CNP with time-domain indices (SDNN, RMSSD, Triangular index) and no association with LF/HF in CKD patients. Our finding may be a reflection of increased CNP production compensating for autonomic dysfunction in the patients. However, a direct effect of circulating CNP on autonomic regulation can also be possible. The exact explanation remains to be fully elucidated.

Due to increased stimuli and possible reduced renal clearance, plasma natriuretic peptides, including ANP and BNP are generally elevated in patients with impaired kidney function [22–24]. However, in our study, we did not observe an association between plasma CNP and eGFR in patients with CKD. The absence of an association between CNP and eGFR in our study is consistent with a previous report by Sangaralingham et al., in which the authors found no association between CNP and eGFR in the general population [13]. There are two possible explanations for this distinctive characteristic of CNP. First, the production of CNP may be less potent than ANP and BNP in the context of renal dysfunction. ANP and BNP production are mainly stimulated by increased atrial or ventricular wall tension, which is common in CKD patients due to accumulated extracellular fluid volume and elevated blood pressure, whereas CNP expression regulation remains obscure and has been linked to sheer stress on endothelial cells and cytokines [25, 26]. However, several studies have noted a negative association between NT-proCNP, the propeptide without biological activity, with eGFR [11, 27–29]. Given that no arteriovenous gradient of NT-proCNP was seen across renal tissue, it seems that CNP production, at least at the propeptide level, is also increased in CKD patients. Second, CNP is cleared in the circulation primarily in a kidney-independent fashion. It can be either hydrolyzed by the neutral endopeptidase or bind to the natriuretic peptide receptor-C to be internalized and degraded [30, 31]. The clearance of CNP in the circulation is very rapid with a half-life of only 2.6 min [32]. This may also explain that why NT-proCNP, but not CNP, is associated with renal function. To be noted, a previous study observed a negative arteriovenous CNP gradient across renal tissue in humans [27]. This implies that the kidney can also contribute to the clearance of CNP, though may to a less important role.

The endothelium is the primary secretion site of CNP in peripheral tissues [33]. Mice with endothelial-specific knockout of the CNP encoding gene, *Nppc*, exhibited elevated blood pressure, as well as endothelial dysfunction and impaired endothelial-dependent vascular relaxation capacity [7, 8]. However, we did not detect a difference in circulating CNP level between patients with endothelial dysfunction and those without. Furthermore, there is also no association of plasma CNP with blood pressure or pulse wave velocity. The lack of a link between circulating CNP and measurements of vascular homeostasis in our study seems to support the questioned notion that the hemodynamic regulation actions of CNP are paracrine or autocrine, instead of endocrine [19, 34]. This is in line with the findings that polymorphism of the CNP gene was an independent predictor of development of hypertension in a Japanese population but plasma CNP was not increased in hypertensive subjects compared with normotensive controls [18, 35].

Several limitations of our study should be pointed out for proper interpretation of our results. First, due to the cross-sectional design, we cannot draw any causative conclusion. Second, our findings may be somewhat limited by the small sample size and need to be confirmed in population-based studies. The lack of healthy controls in the current study leaves the question that whether CNP level differs between individuals with and without CKD unsolved. Third, more detailed cardiac evaluations, e.g. echocardiography or cardiac injury biomarker measurements, were lacking in our study, limiting our ability to explore the association of CNP with cardiac function and injury. Nevertheless, our study remains a first step in evaluating the link between circulating CNP and the cardiovascular system in patents with CKD and can offer indications for further exploration.

## Conclusions

In summary, in a group of patients with CKD, reduced renal function was not associated with elevated plasma CNP. Circulating CNP showed no association with blood pressure, endothelial function or arterial elasticity. There was a significant association between elevated CNP with reduced HRV. Our data highlights a possible link between circulating CNP and autonomic dysfunction in CKD patients. Further studies are warranted to explore the mechanisms underlying this association, as well as evaluate the ability of circulating CNP in predicting adverse cardiovascular event in CKD patients.

## Additional file

Additional file 1: **Table S1.** Sensitivity analysis excluding patients with coronary artery disease. (DOCX 17 kb)

## Abbreviations

ANP: Atrial natriuretic peptide
BNP: B-type natriuretic peptide
cfPWV: Carotid–femoral pulse wave velocity
CKD: Chronic kidney disease
CNP: C-type natriuretic peptide
crPWV: Carotid–radial pulse wave velocity
eGFR: Estimated glomerular filtration rate
HRV: Heart rate variability
RHI: Reactive hyperemia index
