# Supplementary material for: Increased circulating bioactive C-type natriuretic peptide is associated with reduced heart rate variability in patients with chronic kidney disease
Source: BMC Nephrol. 2018 Mar 5;19:50. doi: 10.1186/s12882-018-0843-3 (PMC5839007; doi:10.1186/s12882-018-0843-3)
Supplement: Supplementary file 1 — Table S1. Sensitivity analysis excluding patients with coronary artery disease. (DOCX 17 kb) [file 12882_2018_843_MOESM1_ESM.docx]

Additional file 1

Table S1. Sensitivity analysis excluding patients with coronary artery disease

|  | Plasma CNP | | |
| --- | --- | --- | --- |
|  | ß | 95% confidence interval | *p* |
| SDNN* | -0.11 | -0.19 – -0.03 | **0.006** |
| RMSSD* | -0.11 | -0.20 – -0.01 | **0.03** |
| Triangular Index* | -0.08 | -0.14 – -0.03 | **0.005** |
| LF/HF* | 0.03 | -0.09 – 0.14 | 0.65 |

* These parameters were logarithm transformed in the analysis due to their skewed distribution.

ß indicates per SD increment of CNP and was adjusted for age, sex, eGFR, diabetes mellitus, body mass index, current smoker, previous history of cardiovascular diseases, hemoglobin and systolic blood pressure.

Bold entries indicate statistical significance.
